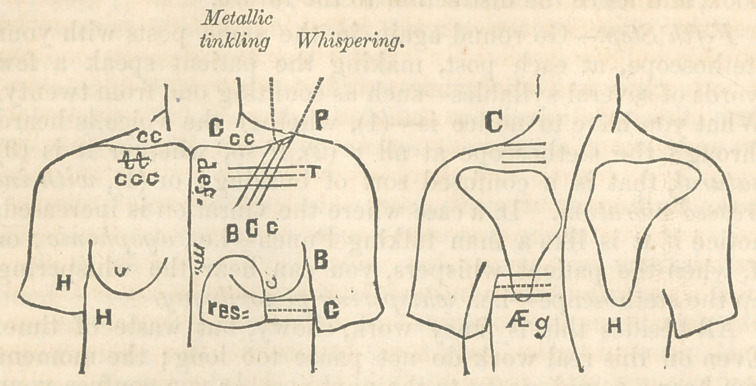# “Drill for Auscultation”—No. 1

**Published:** 1861-09

**Authors:** Thomas K. Chambers

**Affiliations:** Censor of the College of Physicians, Physician to St. Mary’s Hospital and Lecturer on Medicine at St. Mary’s Medical School


### "DRILL FOR AUSCULTATION."—No. 1.

By THOMAS K. CHAMBERS, M. D.,

Censor of the College of Physicians, Physician to St. Mary's Hospital and Lecturer on Medicine at St. Mary's Medical School.

*Reprinted from the London Lancet.*

In all books about Auscultation the necessity for filling a volume, forces the authors to insert a quantity of extraneous matter, by which the real points are smothered. Students are deterred by the formidable look of the subject. But in reality there is no aid to diagnosis or guide to treatment which requires less scientific study or less special tact in its employment. Shelve your books and study nature. All that is wanted is a certain amount of business-like method, common sense, straightforward eyes, and open ears. Use the following order of drill, and I engage that in three or four days you will be capable of reporting on any case that comes before you—you will have acquired all that is practically useful in the clinical examination of the lungs:—

*First Step of Drill.*—Look over the whole outside of the chest, and see if there is anything wrong about the *skin* or deformity of *shape*. Note particularly *flatness or bulging* of ribs. In males this is quickest done by stripping the upper part of the body all at once. A female's shift is looser, and can be drawn *first backwards* so as to look down the back and sides, *then forwards* to look at the front and sides.

*Second Step.*—Get behind the patient, put your two thumbs on the top of the two scapulæ, and lay your fingers firm and flat on the collar-bones and upper ribs. Make the patient sigh deeply, and then looking along your fingers you can compare the motion of the two *upper lobes* of the lungs with one another. If a bright light shines on your finger-nails, you can detect a difference in expansion of one-twentieth of an inch. Then put your thumb-tips on the lowest dorsal vertebra and span the waist as far as you can, make the patient sigh, and you feel the extent of motion of the *lower lobes.*

*Third Step.*—Percussion—what do you want to find out by it? Only if one part of the pulmonary tissue is more solid than another. Of course it cannot be *all* solid; so if any part is so, it will contain less air, and sound therefore, less drummy, less "resonant" than a more healthy part with which you compare it. Do not think about finding anything else, or you will not pay due attention to this.

Place the patient so that you can lay the fingers of your left hand flat on the thorax. Swing the right hand free and easy, and hammer with your finger-*tips* on your knuckles in the following order:

AUSCULTATION POSTS.

| | |
|---|---|
| 1. On right clavicle. | 7. Close uuder right scapula. |
| 2. On left clavicle. | 8. Close under left scapula. |
| 3. Under right clavicle. | 9. Under right mamma (inch below). |
| 4. Under left clavicle. | 10. Under left mamma. |
| 5. Above right scapula. | 11. Right lateral region. |
| 6. Above left scapula. | 12. Left lateral region. |

Do not go on hammering long, but compare by two or three quick strokes each place with the same place on the other side. In all these parts you ought to find the resonance of the two sides equal, except in the left mammary region, where the heart ought to make dull the spot it is felt to beat in.

If you can detect no absence of normal resonance, take a note of it, and go on to next step. But if there be dulness where it ought not to be, percuss round and round the spot where you first find it, and take a note of the *extent of dulness.*

*Fourth Step.*—Apply your stethoscope *flat to the naked skin.* Apply it to the same post in the same order that you have percussed, and make notes in the same order of what you hear. Do not trust to what you may consider deviations from an ideal standard, but compare the two sides and note if they differ.

Now, as to what you may expect to hear:

*Natural Sounds.*—Some healthy lungs breathe very softly, some very harshly: so the degree of sound, if equal throughout, tells nothing. Note therefore, only when they are *defective* in any places, while breathing fully elsewhere.

*Morbid Sounds.*—Think first what your ear *can* tell you, and do not trouble your brains by thinking about what it *cannot.* It can tell you—

(*Point* 1) Whether air enters the subjacent lung or not;

(*Point* 2) Whether it passes through natural, soft, yielding tubes, or through stiffened tubes;

(*Point* 3) Whether it bubbles through fluid or not;

(*Point* 4) Whether the bubbles are large or small, and therefore whether the open spaces they break in are large or small.

*As to Point* 1—if no air enters, there is an end of your observation at that spot; take a note and go on.

*As to Point* 2—if the tubes and tissues are natural, the sound of expiration is less than the sound of inspiration. If it is equal to it or greater than it at any spot compared with the opposite side, call it "tubular breathing"—that is to say, the air goes through tubes only, and does not enter into the terminal vesicles. The typical example of it is what you hear on each side of the sternum over the large bronchi, and therefore it is often called "bronchial breathing." Tubular breathing tells you that the tubes are stiffened either by their coats being swelled or hardened, or by the surrounding tissue being condensed.

*As to Point* 3—air passing through a thickish fluid, like mucus or pus, is sure to make a crackling noise. If it does not do so, if there are continuous whistling, snoring, or piping sounds, they may be safely called "*dry*" sounds and noted down as "whistling," "snoring," or "piping," without waiting to find a French name for them.

*As to Point* 4—the size of the crackles is important. The very finest are evidence of their being situated in the terminal vesicles of the lung. Imitate this fine crepitation by rubbing your hair between your fingers, and then make as large a bubble as you can with saliva between your lips, and you hear the types of the two ends of the scale of size. The larger they are, the larger the bronchus or cavity in which the bubbles break.

I said that air passing through the viscid fluid is sure to make a crackling noise, so that the *absence* of crackling proves the absence of fluid. But the *presence* of crackling does not necessarily prove the presence of fluid. Unfortunately animal membranes will make a crackling just as leather does. And the crackling of inflamed pleura exactly resembles the crackling of small bubles. *The sounds themselves* are indistinguishable. Do not be taken in by persons who profess to distinguish them; they do it by collateral circumstances, as you must do. When you hear crackling, put it down in your note-book, and leave the distinction to the future.

*Fifth Step.*—Go round again in the same posts with your stethoscope, at each post, making the patient speak a few words of several syllables—such as counting one from twenty. What you have to notice is—(1), whether the voice is heard through the stethoscope at all; (2), if so, whether it is (3) *natural*, that is, a confused sort of buzzing; or (4), *with increased vibration.* In a case where the vibration is increased, notice if it is like a man talking Punch—i.e., *ægophonic;* or if, when the patient whispers, you can hear the whispering up the stethoscope—i.e., *whispering pectoriloquy.*

All besides this is fancy work, showy, but waste of time. Even on this real work do not pause too long; the moment you hear a sound, go on to the next post, or you confuse your ear and learn to dawdle.

I will in the next lecture, show you a quick way of taking private notes, and then sketch out a system of estimating the information afforded by what you have heard, and basing a diagnosis upon it.

---

### No. II.

It is a great saving of time in practice to be able to take notes with rapidity. For this purpose I recommend you to acquire the habit of drawing a rough but correct outline of the chest and shoulders, and to have certain marks to record what you have observed. To do it, occupies a quarter of the time of a written description. I make the two outlines which

RIGHT. LEFT. LEFT. RIGHT.

I show you here on the slate in fifty seconds, and in thirty seconds more I can mark upon each of the posts of ascultation what I hear by these or any other arbitrary signs.

In this sketch the most commonly observed phenomena are recorded by conventional signs, the less common by the first few letters of their names, the still less common by the name written at full length.

A curved line along the spine, (, may denote that the spine is curved to one side or another; and another that there is bulging of the ribs.

Oblique lines, ///, that there is flatness of the chest observed, the first step of the drill.

Parallel lines, ≡, that there is dulness on percussion.

*T*, or *t*, that there is tubular breathing; the loudness and depth of tone, being indicated by the size of the letter, showing the size of area it is made in.

*C*, or *c*, or *cc*, that there is crackling, large or small, according to the size of the letter, and extensive according to the number of the letters.

*B* may denote bronchial or vibratory voice.

*P*, pectoriloquy.

*Def. m.* may be written on the line to denote *deficient motion* of the ribs as far as that line extends.

*Æg.* may denote *ægophony ; res.*, *unnatural resonance*, with a line drawn around it to show how far it extends ; while "*whispering voice*," "*metallic tinkling*," "*cracked pottery*," or any fancy sound which you think it worth while to record, had better be written at full length, with a dotted line leading from the post where it is heard.

*H* of course means *healthy sounds*, which you should take a note of, though they do not necessarily prove that the lung is healthy. Remember, now and always, that your note records what you hear, not your diagnosis. Thus on the diagram you may see I have noted that in the left infra-cavicular region the ribs are flattened, though the clavicle has not fallen in; that there is a lateral curvature of the spine, with the greatest projection towards the left side; that the ribs on the left side bulge considerably; that there is a deficient motion of both upper and lower lobes of the lungs on the left side; that there is dulness on percussion in the left infra-clavicular, left supra-scapular, and left lateral regions; that the cardiac region is resonant; that there are crackling or crepitant râles in the right supra-clavicular and left supra-clavicular regions; that in the latter these are the larger and coarser; that there are the same râles in greater amount and with small tubular breathing in the right infra-clavicular; that in the left infra-clavicular there is also very loud tubular breathing, large and small crepitant râles, very loud bronchial voice, whispering pectoriloquy, and metallic tinkling; that in the left infra-scapular region there is ægophony; that in the left lateral there is bronchial voice and mixed crepitant râles of various sizes; that the sounds were those of a healthy condition of lung in the right infra-scapular, infra-mammary, and lateral regions.

I need not ask you which mode of record you think will take longest or be most exact.

If you are not quick in drawing with a pen, a further saving of time may be effected by having the outline cut in wood for a few shillings, and stamping it in your case-book when and where required. Or you may have it stamped on thin paper, gummed at the back like a postage stamp, and stick it in when wanted.

Under any circumstances, whether you adopt my method or one of your own, I think it of paramount importance that you should take notes, not of your diagnosis, but of the *ground on which you base it.* It is of no use to yourself, your patient, or to science, to remember that on such a day you thought that there was pneumonia or tubercle, but it is of great use to all to remenber why you thought so.

---

THE SYLVESTER METHOD.—Our British brethren are gradually superseding the ready method of Marshall Hall for restoring suspended animation, by the still simpler and readier one of Dr. SYLVESTER, which is thus given in Heath's *Minor Surgery:* Lay the patient on his back, and having pulled the tongue forward, draw the arms slowly up over the head, by which means the ribs are elevated by the pectoral muscles, and inspiration is produced; the arms are then to be brought down to the side of the chest, which they are to compress in a slight degree. These movements are to be repeated as slowly as in the other method (the Marshall Hall method), and it is said that they give a more complete charge of air to the lungs.—*Boston Med. and Surg. Journal.*

## Figures and Tables

**Figure f1:**
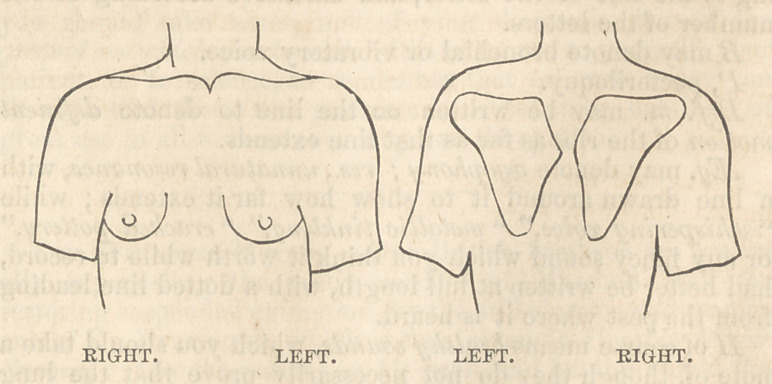


**Figure f2:**